# A network-based meta-analysis for characterizing the genetic landscape of human aging

**DOI:** 10.1007/s10522-017-9741-5

**Published:** 2017-12-21

**Authors:** Hagen Blankenburg, Peter P. Pramstaller, Francisco S. Domingues

**Affiliations:** 1 Institute for Biomedicine, Eurac Research, Affiliated Institute of the University of Lübeck, Viale Druso 1, 39100 Bolzano, Italy; 2 Department of Neurology, General Central Hospital, Bolzano, Italy; 3 Department of Neurology, University of Lübeck, Lübeck, Germany

**Keywords:** Human aging, Meta-analysis, Age-related disease, Network analysis, Protein complex, Network cluster

## Abstract

Great amounts of *omics* data are generated in aging research, but their diverse and partly complementary nature requires integrative analysis approaches for investigating aging processes and connections to age-related diseases. To establish a broader picture of the genetic and epigenetic landscape of human aging we performed a large-scale meta-analysis of 6600 human genes by combining 35 datasets that cover aging hallmarks, longevity, changes in DNA methylation and gene expression, and different age-related diseases. To identify biological relationships between aging-associated genes we incorporated them into a protein interaction network and characterized their network neighborhoods. In particular, we computed a comprehensive landscape of more than 1000 human aging clusters, network regions where genes are highly connected and where gene products commonly participate in similar processes. In addition to clusters that capture known aging processes such as nutrient-sensing and mTOR signaling, we present a number of clusters with a putative functional role in linking different aging processes as promising candidates for follow-up studies. To enable their detailed exploration, all datasets and aging clusters are made freely available via an interactive website (https://gemex.eurac.edu/bioinf/age/).

**Electronic supplementary material:**

The online version of this article (10.1007/s10522-017-9741-5) contains supplementary material, which is available to authorized users.

## Introduction

Age is an important risk factor for a number of diseases (Cutler and Mattson 2006; Niccoli and Partridge 2012). A better understanding of the connections between the diverse aging processes and the onset and progression of age-related diseases is expected to have an impact on individual health span and population health (Demetrius and Fraifeld 2014). A common foundation of the complex aging-related changes on a cellular and molecular level has been proposed via the nine hallmarks of aging (López-Otín et al. 2013), but while key mechanisms and processes have been identified, their molecular foundations remain largely uncharacterized (Kenyon 2010). At the same time technological advances and falling costs have enabled large-scale assessments of a wide range of aging processes and have led to a great number of publicly available *omics* datasets (de Magalhães and Tacutu 2016). One area that has been intensely studied in recent years are aging-related changes in DNA methylation, an epigenetic process associated with controlling gene expression (Jones et al. 2015). The studies observed a genome-wide decrease in the methylation of cytosine guanine dinucleotides (CpGs) with increasing age, while a number of gene promoters were increasingly methylated in a tissue-dependent manner (Teschendorff et al. 2013). More specifically, the methylation status of certain CpG sites in the genome was found to be predictive for the chronological age of a person (Hannum et al. 2013; Horvath 2013) and was also linked to biological age and overall lifetime mortality (Marioni et al. 2015). The limited overlap between individual DNA methylation studies, however, requires the joint analysis of multiple datasets in order to fully interpret partial and potentially contradicting results. For example, two meta analyses reported 11 and 41 high-confidence CpG markers, respectively, that were found in at least four different studies and identified common biological processes and cellular pathways of differentially methylated genes (Steegenga et al. 2014; Jones et al. 2015).

Interaction networks, in which genes and their products are represented as nodes, which are connected by edges that represent different relationships, are valuable tools for integrative data analysis and for identifying disease associations (Barabási et al. 2011; Menche et al. 2015). Integrative network-based analyses have also been performed in aging research [reviewed in (Simkó et al. 2009; Peysselon and Ricard-Blum 2011; de Magalhães and Tacutu 2016)]. Notable example applications identified highly connected and important hub proteins (Sőti and Csermely 2007; Wolfson et al. 2009), reported connections between longevity-associated genes (Budovsky et al. 2007), investigated links between aging genes and age-related diseases (West et al. 2013b; Fernandes et al. 2016), and made network data available as part of dedicated aging databases (de Magalhães et al. 2005; Tacutu et al. 2010). West et al. developed a network clustering algorithm and applied it to the integration of DNA methylation datasets, identifying a number of clusters that were enriched in stem-cell differentiation pathways (West et al. 2013a).

In the present study we extended this approach in various ways and performed the largest and most diverse meta-analysis of human aging genes. In particular, by using substantially larger networks and incorporating more DNA methylation and other aging-related datasets than any previous study, we were able to uncover novel gene-aging associations, which were made available via an online resource for easy exploration.

## Results

### The largest and most diverse compilation of human aging genes

**Fig. 1:**
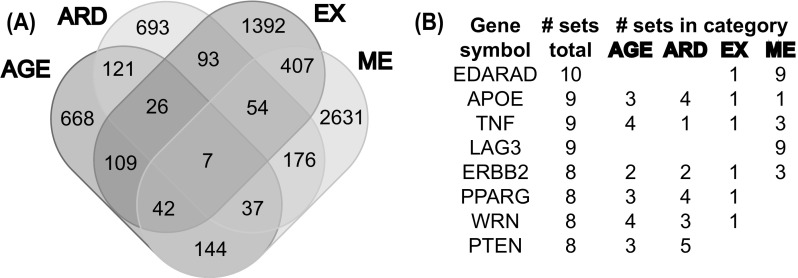
**a** Gene-based overlaps of all aging dataset categories. AGE: curated aging; ARD: age-related disease; EX: gene expression; ME: DNA methylation. **b** List of genes with eight or more aging dataset associations

The meta-analysis presented here unifies 35 datasets related to a wide range of aging aspects, grouped into the four categories DNA methylation changes (ME), gene expression changes (EX), age-related diseases (ARD), and curated aging data (AGE). In total, associations with 6600 human genes are reported, with 3498 genes in sets of the ME category, 2130 in EX, 1207 in ARD, and 1154 in AGE (Table 1). The great majority of genes are only reported in a single category (Fig. 1a), 1050 genes are associated with two, 159 genes with three, and seven genes with all four categories. Those seven genes are APOE (apolipoprotein E), CCT7 (chaperonin containing TCP1 subunit 7), ERBB2 (erb-b2 receptor tyrosine kinase 2), PRKCA (protein kinase C alpha), RASSF1 (Ras association domain family member 1), SREBF1 (sterol regulatory element binding transcription factor 1), and TNF (tumor necrosis factor). APOE, ERBB2, and TNF are also among the genes associated with the highest number of aging datasets (Fig. 1b). Two other genes in this list are EDARADD (EDAR associated death domain) and LAG3 (lymphocyte activating 3), which have the strongest overall evidence for aging-associated DNA methylation changes with reports in nine different ME datasets. Notably, of the 117 genes that are reported in four or more ME sets, the criterion used in previous studies for selecting high-confidence markers, 87 genes are exclusively annotated to the ME category and have no additional association with any other aging dataset.

**Table 1:** Aging datasets used in this analysis

Set name	Reported genes	Reference
8 curated aging sets (AGE)	1154 curated aging genes	
AGE_Chaperones	88	Brehme et al. (2014)
AGE_Co_Chaperones	244	(Brehme et al. (2014)
AGE_GenAge	100	Tacutu et al. (2013)
AGE_GenAge_Indirect	198	Tacutu et al. (2013)
AGE_Longevity	195	Tacutu et al. (2013)
AGE_Longevity_HT	144	Tacutu et al. (2013)
AGE_Senescence	342	Zhao et al. (2016)
AGE_mTOR	60	Kanehisa et al. (2016)
10 age-related disease sets (ARD)	1207 age-related disease genes	
ARD_HGMD_Cancer	226	Stenson et al. (2014)
ARD_HGMD_Cardio	402	Stenson et al. (2014)
ARD_HGMD_Diabetes	83	Stenson et al. (2014)
ARD_HGMD_Neuro	34	Stenson et al. (2014)
ARD_HPO_Ageing	126	Köhler et al. (2014)
ARD_HPO_Cancer	427	Köhler et al. (2014)
ARD_HPO_Cardio	164	Köhler et al. (2014)
ARD_HPO_Diabetes	28	Köhler et al. (2014)
ARD_HPO_ Neuro	74	Köhler et al. (2014)
ARD_HPO_Stroke	34	Köhler et al. (2014)
4 gene expression sets (EX)	2130 differentially expressed genes	
EX_Magalhaes	73	de Magalhães et al. (2009)
EX_Mercken	485	Mercken et al. (2013)
EX_Peters	1497	Peters et al. (2015)
EX_Sood	153	Sood et al. (2015)
13 DNA methylation sets (ME)	3498 differentially methylated genes	
ME_Bacalani	44	Bacalini et al. (2015)
ME_Bell	444	Bell et al. (2012)
ME_Bocklandt	81	Bocklandt et al. (2011)
ME_Florath	122	Florath et al. (2014)
ME_Hannum	117	Hannum et al. (2013)
ME_Heyn	1445	Heyn et al. (2012)
ME_Horvath	344	Horvath (2013)
ME_Marttila	239	Marttila et al. (2015)
ME_Rakyan	138	Rakyan et al. (2010)
ME_Steegenga	436	Steegenga et al. (2014)
ME_Teschendorff	591	Teschendorff et al. (2010)
ME_Weidner	105	Weidner et al. (2014)
ME_Xu	679	Xu and Taylor (2014)
35 aging sets in total (ALL)	6600 distinct genes associated with aging

More than two-thirds of all 6600 aging-associated genes are only reported in a single dataset (Table S1). The percentage of overlapping genes between datasets is generally low (Fig. S1), and datasets with significant overlaps are usually from the same category. In the AGE category examples are the curated mTOR pathway (AGE_mTOR), where about half of the genes are also reported in the longevity database (AGE_Longevity), in the ARD category the datasets from the Human Gene Mutation Database (HGMD) and the Human Phenoype Ontology (HPO) that capture related diseases, such as ARD_HPO_Stroke and ARD_HGMD_Cardio, and in the ME category the datasets ME_Weidner and ME_Bocklandt, where about one-third of the genes are also reported in the datasets ME_Teschendorff and ME_Xu. There are very few overlaps between datasets in the EX category or between datasets from different categories, the most notable exception are the DNA methylation changes reported in ME_Martilla, which are largely covered by the gene expression changes reported in EX_Peters.

**Fig. 2:**
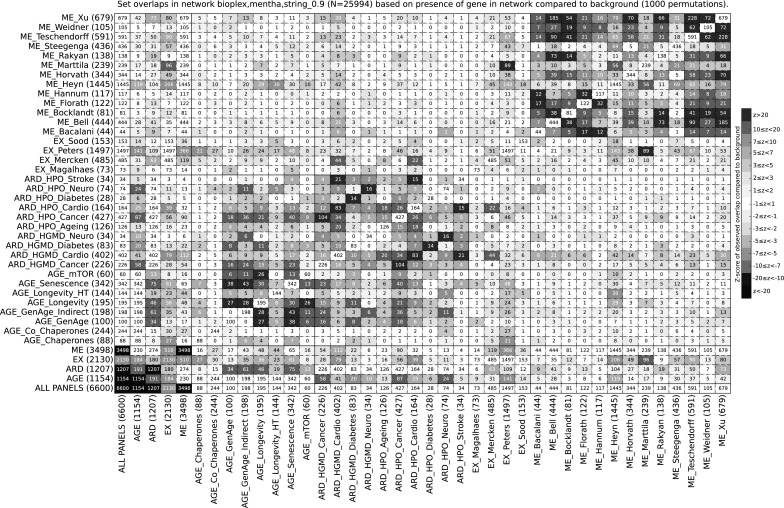
Pairwise gene-based overlaps of all aging datasets. The 35 aging datasets and the four dataset categories are listed on both axes, intersecting cells list the number of overlapping genes. Cells are colored with a blue–white–red gradient that represents the z-score of the observed overlap compared to a randomized background distribution, with shades of blue representing negative z-scores, white z-scores around 0, and shades of red positive z-scores. (Color figure online)

To assess if the observed dataset overlaps are different from those that would be expected when comparing datasets of these particular sizes, they were compared to a randomized background distribution (see “Materials and methods” for details). A great number of dataset overlaps were significantly larger than what would be expected, including all the abovementioned examples (Fig. 2). The most significant overlaps were found within the ME, AGE, and ARD categories, but also between datasets of the AGE and ARD categories. Examples for the latter are the overlaps between the two cancer sets (ARD_HGMD_Cancer and ARD_HPO_Cancer) and the majority of curated aging sets, the caloric restriction gene expression markers reported in EX_Mercken and the two cardiovascular disease sets (ARD_HGMD_Cardio and ARD_HPO_Cardio), and the methylation markers reported in ME_Heyn, which significantly overlap with the two longevity sets AGE_Longevity and AGE_Longevity_HT.

### Aging datasets are well connected through network neighborhoods

To identify further connections between all aging genes and datasets, they were then incorporated into a combined human interaction network composed of 371,847 interactions between 17,451 genes. Only genes with at least one reported interaction were included in the network, which was the case for 5949 of the 6600 aging-associated genes. Different metrics allowed for assessing how central or important a node in this network is. The node degree, for example, lists the number of direct interaction partners; betweenness centrality indicates how many of all the shortest paths between all the nodes in a network pass through a certain node. The higher network degrees and betweenness centralities of the aging-associated genes (Fig. S2, Fig. S3) in general confirmed that they occupy more central and important positions in the combined human interaction network. Aging genes with the lowest degrees are in datasets from the ME and EX categories, while genes reported in the ARD and in particular the AGE category have degrees that are substantially higher than the average background. The highest degrees are found in genes from the curated AGE_GenAge and AGE_GenAge_Indirect datasets, followed by AGE_Senescence and AGE_mTOR. The outliers in the AGE category, which contain genes with average degrees close to the overall background, are the datasets AGE_Longevity_HT and AGE_Co_Chaperones. It is notable that a number of datasets such as AGE_HPO_Stroke and ARD_HGMD_Neuro have mean degrees and betweenness centralities that are considerably higher than their medians, which indicates that these datasets contain few genes that are exceptionally well connected hubs or that are involved in a great number of shortest paths.

### The landscape of network clusters involved in human aging

**Fig. 3:**
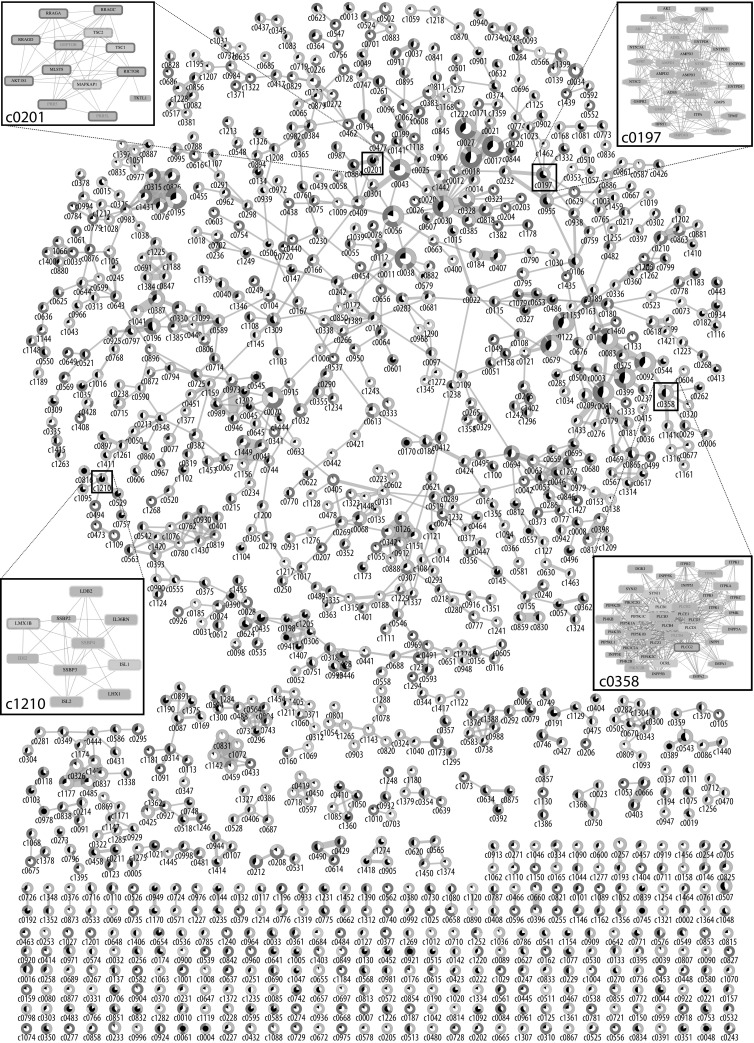
Landscape of all 1079 human aging clusters in the combined interaction network. The aging clusters are depicted as nodes in the main network, edges indicate genes that are shared between the connected clusters. Node size and edge width represent the cluster size and the number of shared genes, respectively. Each cluster is filled with two circles: the inner circle represents the percentage of aging genes in the cluster, ranging from a small black slice (one gene) to full black (all genes in the cluster), the outer circle describes the categories represented by those aging genes, with red for curated aging (AGE), yellow for age-related disease (ARD), blue for gene expression (EX), and violet for DNA methylation (ME). The four boxes provide visualizations of selected aging clusters, where nodes represent genes and edges represent physical protein interactions, protein co-complex relationships, or functional associations. Genes without an aging association are depicted as rectangles with grey fill color, no border, and black text labels. Genes in the AGE category have a solid red border, ARD genes yellow fill color, EX genes blue text color, and ME genes a violet dotted border; genes reported in an AGE and ME set have a red dotted border. Drug targets are depicted as octagonal nodes. All clusters can be visualized and interactively explored at our web resource. (Color figure online)

Network clusters are parts of the network where the nodes have more interactions with other nodes from the cluster than they have with neighboring nodes outside of it. Depending on the edge relationship captured in the underlying network, these clusters might represent proteins that work together in complexes or that participate in a common biological process. The combined human protein interaction network used in this study contains 1263 clusters composed of four or more genes (see “Materials and methods” for details). Of these, 1079 contain at least one and 803 clusters at least two aging-associated genes (Table S2). The clustering algorithm used in this study enables individual genes to be part of multiple clusters. The resulting overlaps can be observed in the visualization of the landscape of all human aging clusters (Fig. 3), where 885 clusters share between one and 64 genes with another cluster and 610 of those form one large connected component.

A web resource was developed to provide access to all 35 datasets and visualization of the 1079 aging clusters (https://gemex.eurac.edu/bioinf/age/). Example visualization created with this resources are the four boxes in Fig. 3, which represent selected network clusters that either capture known aging processes (c0201) or provide promising candidates for follow-up inspections based on a combination of aging associations, enrichment in relevant biological processes or the presence of drug targets (c0197, c0358, c1210). Cluster c0201 is almost exclusively composed of genes with a known aging association, with 12 of its 13 genes associated with longevity and eight of those genes reported in the aging-associated nutrient-sensing mTOR-signaling pathway. In contrast, cluster c0197 does not contain a single known or curated aging gene. However, in this cluster, which has strong associations with nucleotide metabolic processes, two-thirds of the 30 genes are annotated to a set from the ARD (one), EX (13), or ME (8) category and 13 genes are targets of approved drugs. Cluster c1210 includes genes involved in organism development and cell differentiation. In particular, nine out of its ten genes are annotated to an aging set, combining annotations from 12 different sets that partly confirm each other (e.g. LDB2 is a high-confidence methylation marker reported in the sets ME_Florath, ME_Heyn, ME_Horvath, and ME_Marttila) and partly complement (e.g. IL36RN or LHX1 are only reported in single datasets). In cluster c0358, half of the 42 genes are annotated to 18 different aging sets from all four categories. Notably, this cluster, which is enriched in inositol phosphate metabolic process and signaling, joins 12 genes that are reported in nine different ME sets and additionally connects ARD genes associated with different neurodegenerative and cardiovascular disorders, diabetes, and cancer.

## Discussion

### Limited, but significant agreement between human aging datasets

Limited agreement between aging datasets, in particular those investigating changes in DNA methylation, has been previously reported (Steegenga et al. 2014; Jones et al. 2015). This partial complementarity could be interpreted as lack of data quality, since there are also reports on unreproducible DNA methylation markers (Marioni et al. 2015). However, the differences can also be attributed to variations in study designs and experimental procedures, the sampled cell types and tissues, the methylation arrays, the statistical analysis and normalization techniques, and to the mapping procedures between CpG positions and gene identifiers. In addition, while the overlaps in terms of reported genes are limited, we found that they are very significant compared to a randomized background, in particular between sets of the curated aging (AGE), age-related disease (ARD), and DNA methylation (ME) categories (Fig. 2). In this respect, it is reassuring to observe considerable relative overlaps between most ME sets. From the three exceptions that show lower relative overlaps to other ME sets, two are based not only on DNA methylation but also gene expression data (ME_Steegenga and ME_Marttila) and the third is based on a comparison between just a single newborn to centenarian (ME_Heyn). The lower relative overlap between EX sets can be a result of the different sampling techniques and experimental designs. EX_Peters is by far the largest set, and is based on whole blood expression, EX_Sood is based on gene expression in multiple tissues, EX_Magalhaes is based on multiple datasets from different tissues in different organisms, and EX_Mercken focuses on differential gene expression in muscle tissue upon caloric restriction. In summary, meta-analyses that combine multiple datasets provide a valuable tool for linking partly complementary data and for helping to establish a more comprehensive picture. The study presented here is able to uncover more relationships than any previous approach due to its size and the diversity of datasets that represent a wide range of aging processes.

### Distinct network topologies of aging genes

Interaction networks provide an additional layer for linking proteomic and genomic data. Consequently, integrative network analyses have been used to uncover associations in different applications. An example is guilt-by-association, which is commonly used to infer associations between genes based on known relationships. For example, a gene previously not associated with a certain disease or signaling pathway might be a candidate if it is reported to physically interact with a protein that has a known role in the same disease or pathway. There have been intense debates about the reliability of certain interaction types, for example, reporting that physical protein interactions that have been curated from the published literature are substantially more biased than large-scale experiments (Cusick et al. 2009; Salwinski et al. 2009). In our study we combine different types of interaction networks not only to capture various aspects of the underlying biology, but also to correct for potential shortcomings of individual detection techniques. In particular, we incorporate experimentally determined and literature-curated protein interactions, protein co-complex relationships, and functional associations such as interactions inferred from other species or extracted using text-mining techniques. Overall, the combined human network has more than 370,000 interactions, making it substantially larger than the networks used in previous analyses (West et al. 2013a).

By investigating the topology of interaction networks, genes that occupy central or important positions can be identified. For example, West et al. previously reported that aging genes tend to be located in bridging positions within protein interaction networks, connecting otherwise disjoint parts (West et al. 2013b). We analyzed network topological properties of aging genes and found that node degrees and betweenness centralities of genes in AGE and ARD sets are significantly higher than values of genes in EX and ME sets. Since AGE and ARD datasets largely consist of manually-curated data, while EX and ME sets have mostly been determined on a large- or even genome-wide scale, a possible explanation could be a literature or curation bias: genes that have been studied for historic reasons or that have a disease relevance will continuously receive more attention than genes that are poorly characterized, leading to more reported interactions, more annotations with Gene Ontology terms, or a higher likelihood of being manually curated into specific datasets (Schaefer et al. 2015). In this respect it is noteworthy that AGE_Longevity_HT, which contains results of a single large-scale study, shows degrees and betweenness centralities significantly lower than all other curated datasets.

### Network clusters as robust means for identifying aging hotspots

The differing network topological parameters demonstrate that network neighborhood-based approaches can easily be biased by hub genes with exceptionally high degrees (Wolfson et al. 2009). Network clusters, which represent parts of the interaction network where the nodes are more densely connected with each other than with the surrounding regions, offer an additional way for linking genes and for providing functional interpretations (West et al. 2013a; Menche et al. 2015). Importantly, these clusters are more robust to few highly connected outliers, since they are not sufficient to change the overall cluster connectivity (Nepusz et al. 2012). The main characteristic of ClusterONE, the cluster detection algorithm that was used in our study, is that it allows for overlapping clusters, where genes can take part in multiple clusters. In our view this is a more accurate representation of the biological reality than algorithms that separate the network into disjoint groups. In addition, ClusterONE is among the tools with the best overall performance in a recent comparison (Wiwie et al. 2015).

From the more than 1000 computed aging clusters some are very promising candidates for further inspection, either because they contain genes with a strong aging association or because they link a great number of aging datasets. Cluster c0201 (Fig. 3, top-left box) is one example of the former category, capturing many genes of the mTOR signaling pathway, which has been associated with biological age and longevity in a great number of studies and is central to the deregulated nutrient sensing occurring with age (López-Otín et al. 2013). Another cluster with a strong aging-association is c0283. Among its 16 constituting genes is WRN (Werner syndrome RecQ like helicase), one of only two genes that have a direct aging-association in the human GenAge database. Mutations in WRN can cause Werner syndrome, a rare disease associated with premature aging (Gray et al. 1997). In accordance with the increased genomic instability reported in this disease, almost all genes of the cluster are associated with DNA recombination and DNA repair.

The main strength of the data integration approach presented in this study is using network clusters for uncovering new associations between different aging genes and datasets. This is exemplified by cluster c1210 (Fig. 3, bottom-left box). Six of its ten genes are associated with differential DNA methylation in aging, however, since these associations are from six different datasets, the connections would have been overlooked by individual investigations or by combining them in network neighborhoods instead of network clusters. Two of the differentially methylated genes in this cluster encode for enhancers of insulin gene expression and are associated with peripheral nervous system development, which in model organisms has been reported as a regulator of longevity (Wolkow et al. 2000). Another integrative cluster example is c0680, which connects LAG3, one of the two genes reported in the highest number of DNA methylation datasets, with five additional differentially methylated genes and seven genes reported to be differentially expressed in aging. None of the genes in this cluster have yet been curated into an aging database or connected to an age-related disease, although a recent report suggests a connection of LAG3 to Parkinson’s disease (Mao et al. 2016). A more intriguing example is cluster c0197 (Fig. 3, top-right box), which also does not contain any genes previously associated with aging in a curated dataset. However, it links eight genes reported as differentially methylated with age with seven genes reported to have expression changes under long-term caloric restriction, a process that has been found to have a conserved effect on aging in humans and a number of model organisms (Fontana and Partridge 2015). Interestingly, a very recent study in rhesus monkeys reported that caloric restriction delays age-related DNA methylation changes (Maegawa et al. 2017) and clusters such as c0197 could help to unravel the underlying mechanisms. In addition, the availability of multiple approved drugs targeting respective gene products in this cluster provides opportunities for further exploring their potential in improving health trajectories and in the prevention of age-related diseases.

### An online resource facilitating research of human aging genes

Since the number of network clusters with a putative association to aging processes is too large for a detailed exploration in the context of this study, we developed an online resource to make all data publicly available to the biogerontology community. To the best of our knowledge, this is this first resource that provides access to a comprehensive collection of annotated aging clusters, whereas previous studies at most provided source code. All 1079 aging clusters can be sorted by different criteria such as the number of aging genes of a specific category or can be filtered for particular genes of interest. For each cluster an interactive visualization is available, complemented with additional information. For genes this includes Gene Ontology annotations or the information whether it is the target of an approved drug; for interactions this includes links to the source publication or confidence scores. In addition, biological processes are listed that are enriched among the genes in the cluster. To enable further exploration in standalone tools such as Cytoscape, all clusters can be exported as raw networks and high-resolution visualizations in the portable network graphic (PNG) format can be generated. We believe that the resource fosters downstream analysis of aging data and helps to test hypotheses about particular genes of interest.

It is foreseeable that more aging-related large-scale datasets will be made available in the future. Considering the diversity and richness of the data it will remain crucial to provide computational frameworks that aid users in integrating and jointly analyzing such data. We plan to maintain the online resource presented here by updating the underlying network data and functional annotations. We also intend to extend its functionality from the prototypic version to a comprehensive computational aging platform, where users can configure the available data or upload their own data for individual analyses.

## Materials and methods

### Data integration and identifier conversion

Genes and their products are identified using a wide range of identifier systems, such as genomic coordinates, gene symbols, Ensembl and Entrez gene identifiers, or UniProtKB protein accession numbers. To combine the different aging datasets and protein interaction networks, their respective input identifier systems were converted to Entrez gene identifiers. The conversion was done using the Dintor software platform (Weichenberger et al. 2015) with gene mappings for 25,788 unique Entrez identifiers from Ensembl release 75 (Yates et al. 2016). Ambiguous input identifiers that could not be manually resolved were excluded. Entrez gene identifiers that were reported in an aging dataset or an interaction network were included even if they were not present in the Dintor mappings. As a result of one-to-many mappings in the identifier conversion process, some gene numbers mentioned in our study differ from the numbers reported in the original publications. DNA methylation studies that reported CpG positions were mapped to gene identifiers using data from the Bioconductor package IlluminaHumanMethylation450k.db, following the procedures described in the respective publication.

### Aging datasets

The 35 aging datasets listing associations between human genes and various aging aspects are briefly described in the following. Based on the type of data they contain, the sets are grouped into one of the four categories curated aging (AGE), age-related disease (ARD), gene expression (EX), or DNA methylation (ME). All sets are named using a combination of category abbreviation, an underscore, and a short set label, e.g., AGE_mTOR.

### Curated aging (AGE) sets

All data in this category have been manually curated by the respective study authors or database curators. Genes with diverse aging associations in humans and models organisms were retrieved from GenAge (Tacutu et al. 2013). Build 17 was downloaded and separated into gene associations with direct evidence levels (*human*, *mammal*, *model*, *cell*, *human link)* (AGE_GenAge) and those with indirect evidence levels (*functional*, *downstream*, *putative)* (AGE_GenAge_Indirect). Gene associations with longevity were retrieved from build 1 of LongevityMap (Tacutu et al. 2013). The downloaded file was separated into results originating from a single high-throughput study (Sebastiani et al. 2012) (AGE_Longevity_HT) and genes reported in various small-scale studies (AGE_Longevity). The remaining curated aging sets focus on specific hallmarks of aging. The nutrient-sensing insulin/IGF-1-like receptor pathway centered around the mechanistic target of rapamycin (mTOR) is represented by the respective KEGG pathway map (AGE_mTOR) (Kanehisa et al. 2016). Age-related deregulation of cellular proteostasis, a hallmark that is largely maintained by chaperone proteins, is represented by 88 human chaperones (AGE_Chaperones) and 244 co-chaperones (AGE_Co_Chaperones) obtained from the Chaperome Database (Brehme et al. 2014). The aging hallmark of cellular senescence is covered by the literature-curated CSGene database (Zhao et al. 2016). All genes with at least one reported literature reference were included (AGE_Senescence).

### Age-related disease (ARD) sets

The sets in this category contain associations between human genes and different age-related diseases. The associations were retrieved from the commercial Human Gene Mutation Database (HGMD) Stenson et al. (2014) and the Human Phenotype Ontology (HPO) (Köhler et al. 2014). A local MySQL installation of HGMD Professional version 2015.03 was queried using the Dintor platform (Weichenberger et al. 2015), retrieving all genes that were annotated as causative for different cardiovascular diseases (ARD_HGMD_Cardio), neurological disorders (ARD_HGMD_Neuro), diabetes or metabolic syndrome (ARD_HGMD_Diabetes), and cancers (ARD_HGMD_Cancer). Due to HGMD license restrictions only a limited dataset can be made available. HPO gene-disease associations were downloaded from version 2016.01.13 of the HPO ontology browser. The associations were grouped into cardiovascular diseases (ARD_HPO_Cardio), neurodegeneration (ARD_HPO_Neuro), type II diabetes mellitus (ARD_HPO_Diabetes), cancers (ARD_HPO_Cancer), stroke (ARD_HPO_Stroke), and mortality/aging (ARD_HPO_Ageing).

### Gene expression (EX) sets

The four datasets in this category report gene expression changes associated with aging processes or interventions. Genes representing a common gene expression signature of aging (EX_Magalhaes) were obtained from a meta-analysis of multiple studies from mouse, rat, and human (de Magalhães et al. 2009). A meta-analysis of gene expression studies of peripheral whole-blood in humans (Peters et al. 2015) provided genes with different expression profiles in chronological age (EX_Peters). Genes associated with healthy aging (EX_Sood) were taken from a study investigating RNA profiles in aging muscle tissues (Sood et al. 2015). Genes differentially expressed under conditions of caloric restriction were obtained from a study that investigated human muscle biopsies collected from 15 middle-aged individuals practicing long-term caloric restriction and ten age-matched non-obese controls following a normal western diet (Mercken et al. 2013). We analyzed the part of their data that was publicly accessible under Gene Expression Omnibus (GEO) accession GSE38012 with GEO2R (Barrett et al. 2013), using an adjusted *p* value < 0.05 and an absolute expression change z-score > 2 as selection criteria (EX_Mercken).

### DNA methylation (ME) sets

All datasets in this category report age-related changes in the methylation of cytosine guanine dinucleotides (CpGs) in the DNA. The great majority of studies measured the methylation in human blood cells using Illumina 27 K BeadChip or Infinium 450 K arrays. ME_Rakyan contains aging-associated differentially methylated genomic regions that were determined in whole blood of 93 healthy women from 49 to 75 years (Rakyan et al. 2010). Whole blood samples of 261 postmenopausal women provided a DNA methylation signature of aging consisting of 589 CpGs (ME_Teschendorff) (Teschendorff et al. 2010). ME_Bocklandt is based on 88 CpG sites that showed a significant age association in the saliva of 34 male twins (Bocklandt et al. 2011). ME_Bell contains differentially methylated regions associated with age in whole-blood DNA methylation profiles (Bell et al. 2012). Based on a comparison of methylation profiles of a centenarian and a newborn, ME_Heyn contains the subset of the data reporting differential methylation markers found with whole-genome bisulfite sequencing and Infinium arrays (Heyn et al. 2012). ME_Horvath is based on the “aging clock”, a multi-tissue predictor for DNA methylation levels composed of 353 CpG sites (Horvath 2013). ME_Hannum contains 71 CpG markers that predicted chronological age in a study analyzing whole blood of 656 individuals (Hannum et al. 2013). ME_Florath contains 162 CpG sites that were reported with significant age-associations in a cohort of 965 people (Florath et al. 2014). ME_Steegenga lists genes reported with gene expression and DNA methylation changes in peripheral blood mononucleated cells from ten participants (Steegenga et al. 2014). ME_Weidner contains 102 age-associated CpG sites found in human blood (Weidner et al. 2014). ME_Xu contains 749 age-related CpG sites determined in blood samples from women between the age of 35 and 76 (Xu and Taylor 2014). ME_Bacalini is based on a re-analysis of published DNA methylation data from three previous studies (Bacalini et al. 2015). ME_Marttila contains 377 CpG sites found with changes in the DNA methylation and gene expression profiles in blood leukocytes (Marttila et al. 2015).

### Gene annotation and process enrichment

Data from the Gene Ontology (GO) (Ashburner et al. 2000) release 2015.09 were incorporated to identify the biological and cellular roles of all genes. The Dintor software platform (Weichenberger et al. 2015) was used to access all gene annotations from the three GO categories biological process (BP), cellular component, and molecular function, and to compute BP enrichments. In particular, hyper-geometric tests were used to compare the GO terms annotated to the genes in a dataset (e.g., a network cluster) to the terms annotated to the full network background. Multiple testing corrections were done using a false-discovery rate < 0.05 (Benjamini and Hochberg 1995). To determine if genes or their prducts are targeted by approved drugs, data from DrugBank (Wishart et al. 2006) release 5.0.3 were incorporated.

### Interaction networks

The interaction networks used in this study are composed of genes, represented as network nodes, and different types of relationships, represented by edges connecting two nodes. The majority of gene relationships are physical protein interactions or co-complex associations of the encoded proteins, complemented by different types of functional relationships. In particular, the interaction data were retrieved from the resources mentha, BioPlex, and STRING. Human release 2015.11.27 of mentha was used as an integrated interaction network that combines experimentally determined and literature-curated physical protein interactions and co-complexes from a number of different primary interaction databases (Calderone et al. 2013). BioPlex is an ongoing effort to unravel the landscape of all human protein complexes (Huttlin et al. 2015). The initial release v2 is included in mentha, the more complete release v4 was included as a separate network. The STRING database was included as a it combines experimentally determined and literature-curated protein interactions with various types of functional associations, such as interactions inferred from other species, extracted using text-mining techniques, or associations based on gene co-expression (Szklarczyk et al. 2015). Due to potentially spurious functional associations included in the STRING database, only a high-confidence network (string_0.9) was used, which was created by filtering version v10 of the human interaction data and keeping only interactions with a combined confidence score of at least 900 out of 1000, a threshold recommended by the authors. The combined interaction network (bioplex,mentha,string_0.9) was created by integrating the three individual networks.

### Computational resources

All network analyses were computed with Python programs based the Dintor software platform (Weichenberger et al. 2015) and the network library igraph-python (Csardi and Nepusz 2006). Network clusters were determined using the standalone Java version of ClusterONE (Nepusz et al. 2012) with default parameters, adjusting the minimum cluster size to four genes. Connections between network clusters were visualized using the Cytoscape (Shannon et al. 2003) application enhancedGraphics (Morris et al. 2014). The web resource for visualizing network clusters (https://gemex.eurac.edu/bioinf/age/) was implemented using a combination of Hypertext Markup Language (HTML), Cascading Style Sheets (CSS), and JavaScript and has been tested in all major browsers. Interactive network visualizations are rendered using Cytoscape.js (Franz et al. 2016).

### Statistical significance of set overlaps and cluster composition

To determine if the observed gene-based set overlaps are defined by the sizes of the respective datasets, they were compared to a background distribution. This distribution was created by replacing all 35 datasets with randomly sampled datasets of the same size and computing the respective pairwise overlaps. Repeating this process 1000 times provided sufficient background values to compute reliable z-scores for the observed overlap values. In addition, p-values were computed for all clusters using Fisher’s exact test to assess if the number of aging-associated genes were significantly greater than what would expected.

## Electronic supplementary material

Below is the link to the electronic supplementary material.

Supplementary Fig. S1 Gene-based overlaps of all aging datasets (PDF 1816 kb)

Supplementary Fig. S2 Network degrees of all aging datasets in the combined network. (PDF 465 kb)

Supplementary Fig. S3 Network betweenness centralities of all aging datasets in the combined network. (PDF 480 kb)

Supplementary Table S1 List of 6600 genes annotated to any of the 35 aging datasets (XLS 1549 kb)

Supplementary Table S2 All network clusters containing at least one gene from an aging dataset (XLS 531 kb)
