# Supplementary figures and images for: A network-based meta-analysis for characterizing the genetic landscape of human aging
Source: Biogerontology. 2017 Dec 21;19(1):81–94. doi: 10.1007/s10522-017-9741-5 (PMC5765210; doi:10.1007/s10522-017-9741-5)

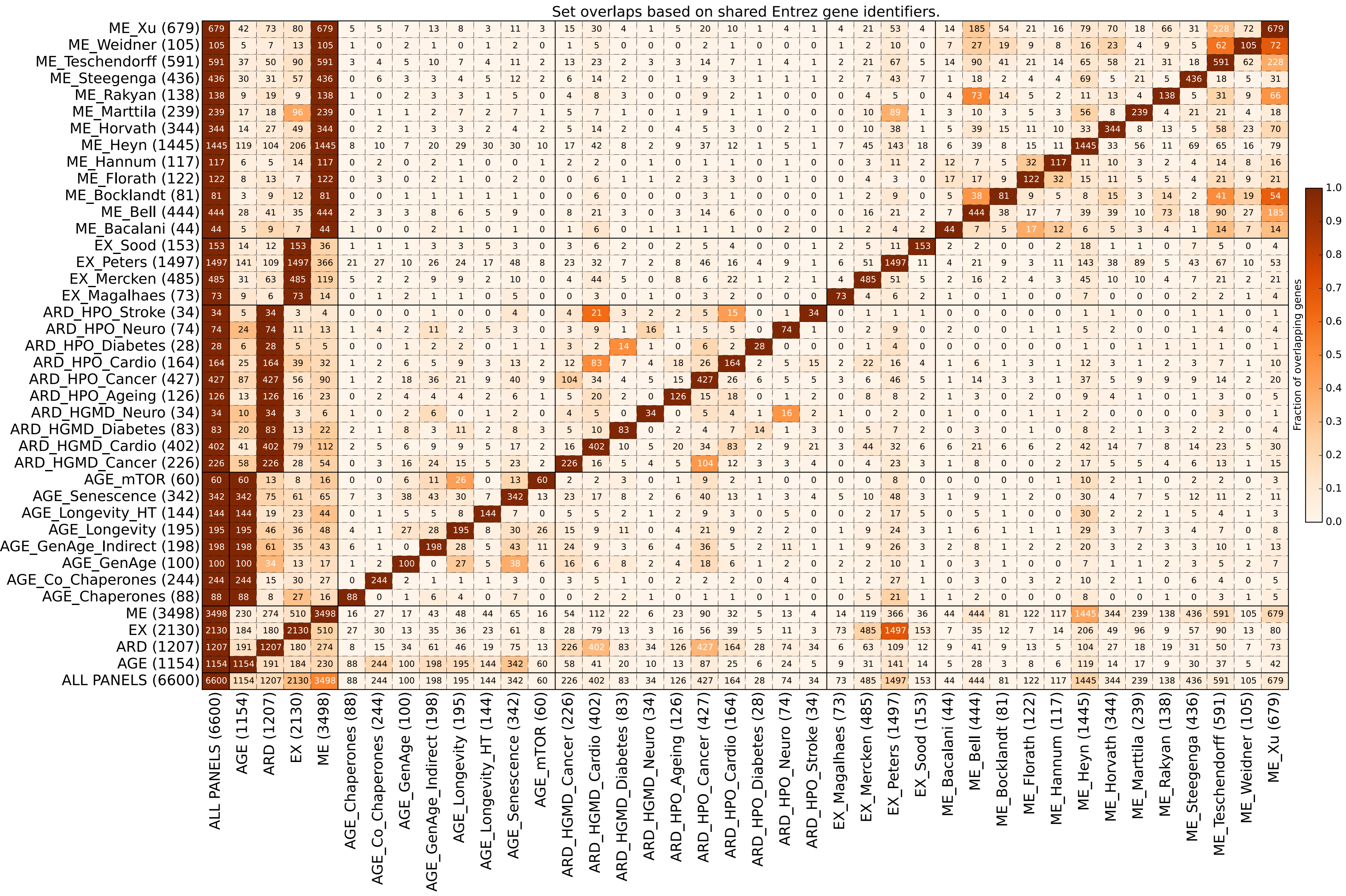

Supplement: Supplementary file 1 — Gene-based overlaps of all aging datasets (PDF 1816 kb) [file 10522_2017_9741_MOESM1_ESM.pdf]

# Network degree bioplex,mentha,string\_0.9 (N=17451)

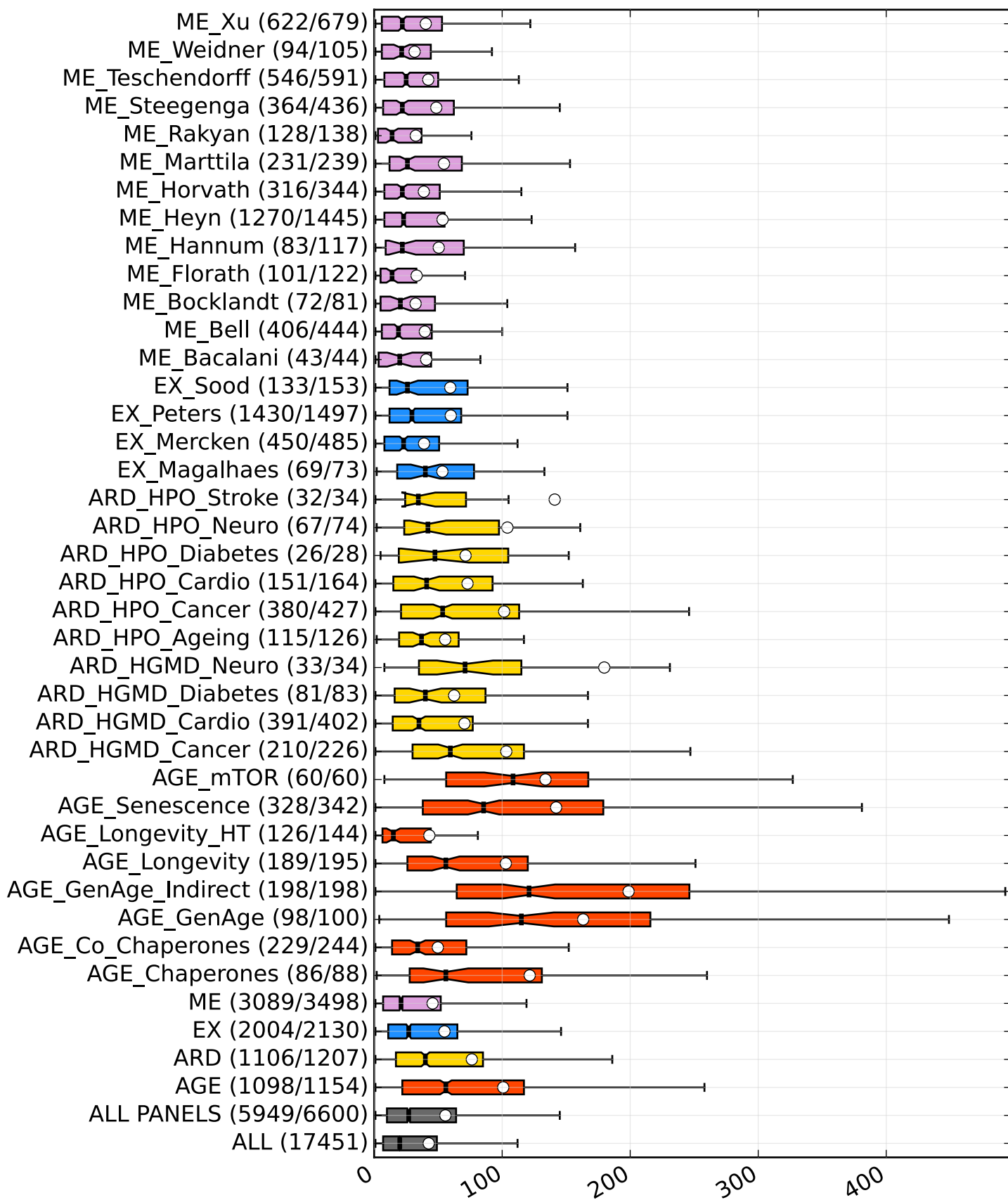

Supplement: Supplementary file 2 — Network degrees of all aging datasets in the combined network. (PDF 465 kb) [file 10522_2017_9741_MOESM2_ESM.pdf]

# Network betweenness bioplex,mentha,string\_0.9 (N=17451)

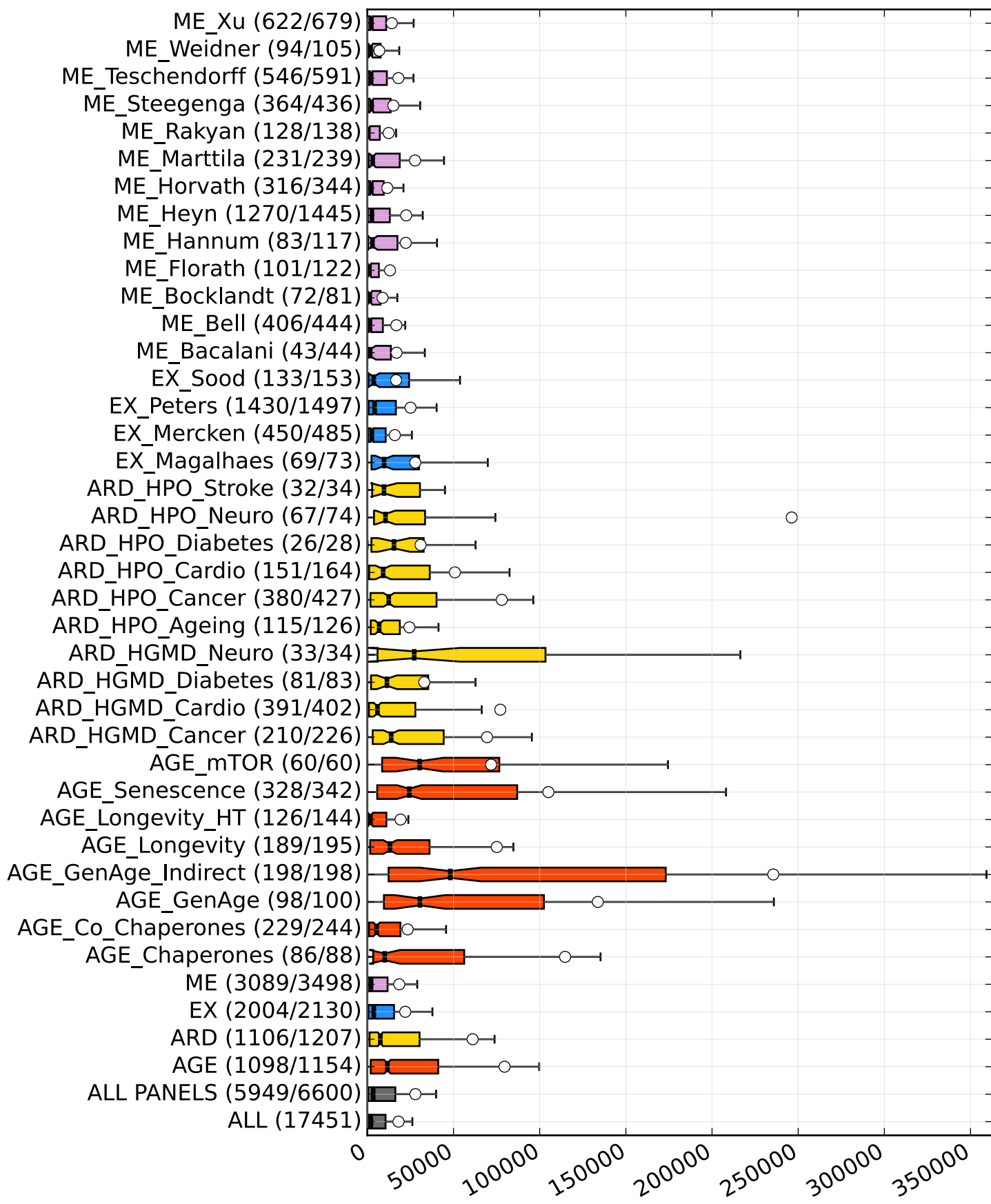

Supplement: Supplementary file 3 — Network betweenness centralities of all aging datasets in the combined network. (PDF 480 kb) [file 10522_2017_9741_MOESM3_ESM.pdf]
